# Radical excision combined with instrumented fixation in the management of thoracic epidural angiolipoma: a case report

**DOI:** 10.1186/1752-1947-8-377

**Published:** 2014-11-20

**Authors:** Yaoki Nakao, Nobuyuki Shimokawa, Yuji Tsukazaki, Aiko Terada, Kosuke Nakajo, Yoshihiko Fu

**Affiliations:** 1 Department of Neurosurgery, Tsukazaki Hospital, 68-1, Waku, Aboshiku, Himeji 6711106, Japan

**Keywords:** Angiolipoma, Radical resection, Spinal epidural tumor

## Abstract

**Introduction:**

Spinal angiolipoma is a benign uncommon neoplasm composed of mature lipocytes admixed with abnormal blood vessels. They account for only 0.04% to 1.2% of all spinal tumors. We present a case of thoracic epidural angiolipoma treated by combining radical resection with instrumented spinal fixation, without any surgical complication.

**Case presentation:**

A 32-year-old Asian woman presented with dorsal epidural angiolipoma at the upper-thoracic level. She had a seven-month history of gradually worsening weakness and numbness in her lower extremities. Imaging studies of her thoracic spine demonstrated a heterogeneously well-enhancing mass, located in her posterior epidural space without surrounding bone erosion at the upper thoracic level. We also observed compression of her thoracic cord. During surgery, a reddish-gray, highly vascularized mass was excised. Her facet joints had to be resected to expose the part migrating into the intervertebral foramen. Because there was concern regarding the stability of her thoracic spine, we performed spinal fixation using pedicle screws. Histopathological study of the surgical specimen showed a typical angiolipoma.

**Conclusion:**

Angiolipomas can be radically excised with good prognosis. Surgical removal is the preferred treatment for spinal angiolipoma, and the prognosis after surgical management is very good. Although outcomes remained favorable despite incomplete resections in a number of spinal angiolipoma, complete removal is preferred. We successfully achieved total resection without any surgical complication by combining radical resection with instrumented spinal fixation.

## Introduction

Spinal angiolipoma is a rare cause of spinal cord compression, accounting for 0.14% to 1.2% of spinal tumors [1,2]. It is a benign tumor of the epidural space, composed of mature fat cells and abnormal blood vessels that vary from capillary through sinusoid or venular to arterial [3]. The most common location is the epidural mid-thoracic area [4,5] and they usually present with slowly progressing signs and symptoms of cord compression [5].

Total surgical resection, and not adjuvant therapy, should be administered to patients with this pathological entity [6]. However, complete removal is not always easy because of anatomical constraints, particularly in cases with infiltration and/or intraforaminal migration.

We present the case of a 32-year-old woman with an upper thoracic dorsal epidural angiolipoma treated by radical resection combined with instrumented spinal fixation.

## Case presentation

**Figure 1:**
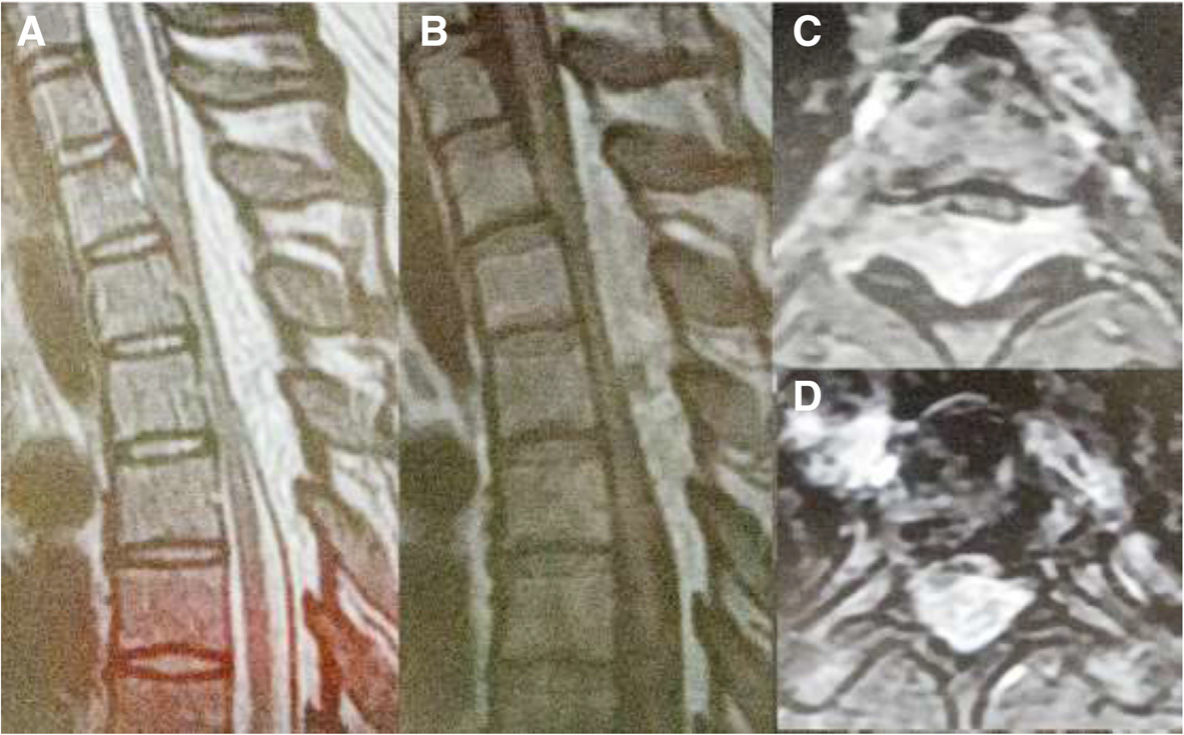
**Pre-operative images. (A)** Pre-operative sagittal T2-weighted magnetic resonance image and **(B)** pre-operative sagittal T1-weighted magnetic resonance image showing a mixed epidural mass at the T1 to T6 level. **(C)** Post-contrast T1-weighted axial magnetic resonance images at the T3 to T4 level and **(D)** T5 level showing the mass along the posterior epidural spinal canal of the thoracic spine, compressing and displacing the spinal cord anteriorly.

**Figure 2:**
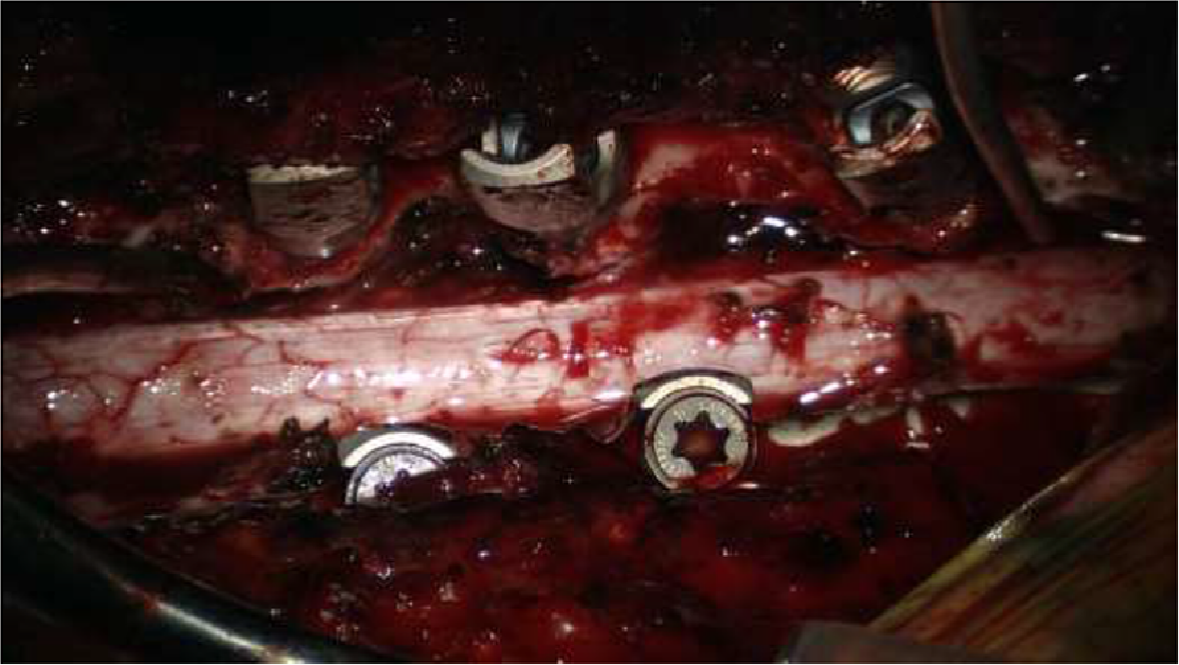
**Intra-operative view.** Intra-operative view of the surgical field showing completely decompressed dural tube, widely resected facet joints, and inserted pedicle screws.

**Figure 3:**
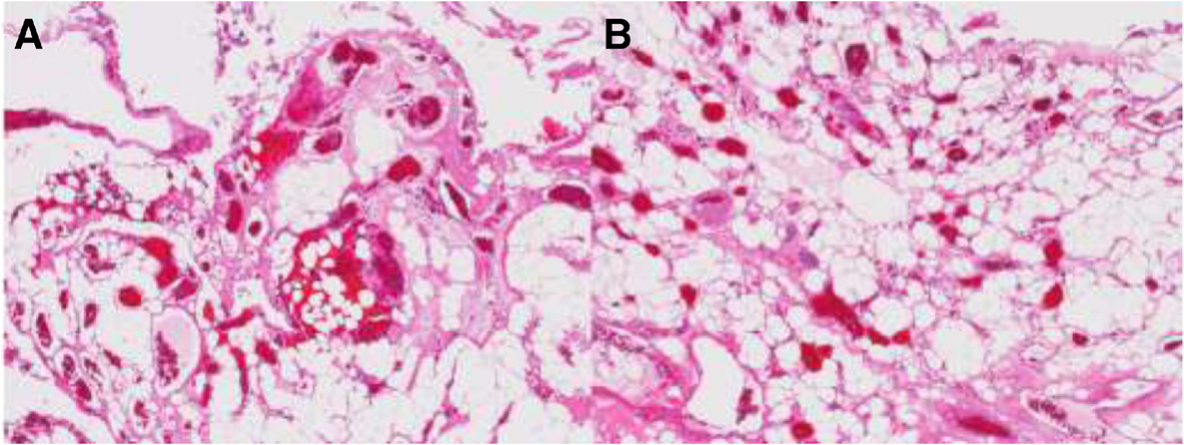
**Pathological findings. (A, B)** The lesion contained mature adipocytes and small- to medium-caliber blood vessels indicative of angiolipoma (hematoxylin and eosin stain ×80).

A 32-year-old Asian woman (height 169cm, body weight 108kg, body mass index 37.8) without any pathological history, such as high corticosteroid intake, presented with gradually progressive weakness and sensory change in her lower extremities over seven months. At the time of presentation, her body mass index had been stable for more than five years. A neurological examination revealed weakness in her lower extremities (manual muscle testing (MMT), grade 3). Her pinprick sensation was moderately decreased (6 out of 10) below the dermatome level of T4 and her vibration sense was decreased in both feet. She had a positive Romberg’s sign. Her deep tendon reflexes were hyperactive on both legs and negative on ankle clonus, straight leg raising, and femoral nerve stretching tests. Bladder-bowel disturbance was evident as pollakiuria. Her pre-operative Japanese Orthopaedic Association (JOA) score for thoracic myelopathy (maximum possible score, 11), derived from the JOA scoring system for cervical myelopathy by eliminating motor and sensory scores for the upper extremity, was 4 (0-1-1-2).Laboratory investigations and plain radiography revealed no abnormalities. A spinal computed tomography (CT) scan revealed that the mass lesion severely compressed her spinal cord in the posterior epidural space at the T1 to T6 level, and the mass extended into the bilateral T2 to T5 neural foramen. There was no obvious bone erosion surrounding the tumor or widened or scalloped neural foramen. Magnetic resonance imaging of the epidural thoracic mass showed hyper-signal intensity on T1- and T2-weighted images and homogenous enhancement with gadolinium (Figure 1). Her spinal cord was compressed and anteriorly displaced.Our patient underwent an operation. A total laminectomy of T1 to T6 using a high-speed drill was performed. Epidural fat was encountered at both the upper and lower end of the lesion. The reddish-gray, highly vascularized mass was below the epidural fat. The well-circumscribed tumor was excised from the dorsal dura mater without difficulty and removed piecemeal while alternately repeating hemostasis by electrocoagulation. After a bilateral T2 to T3, T3 to T4, T4 to T5 facetectomy, we radically excised the extradural mass involving the intraforaminal enlargement. Because of concern regarding the stability of her thoracic spine, we performed spinal fixation using pedicle screws (Figure 2). The removed bone was saved during the surgery and used as a graft to achieve bony fusion. We achieved gross total removal with no additional deficits. Our patient showed an improvement in strength (MMT grade 5) and sensation in the days following the operation. Post-operative imaging showed no residual tumor.On microscopic examination, the resected tumor appeared as an elongated, encapsulated dark red mass. Histological examination revealed a mixture of mature adipose and abundant vascular tissues that ranged from capillary to venular in size, and a few were irregular in shape with thickened walls (Figure 3). Immunohistochemistry staining was positive for cluster of differentiation 34 and smooth muscle actin that were consistent with an angiolipoma. No mitosis was detected according to an examination using Ki-67, a cell cycle marker.

A follow-up neurological examination showed only hyper-reflexia of her lower extremities with no other abnormal findings. Imaging studies six months after the surgery did not show any recurring posterior epidural mass lesions. Her JOA score recovered to 11 out of 11.

## Discussion

Angiolipoma is a rare but benign clinicopathological entity, composed of fatty tissue and vascular elements. It grows in a spindle shape along the spinal canal, without associated vascular malformations. Angiolipoma is considered a distinct clinical and pathological entity, traditionally grouped as a variant of lipomas. Characteristically, the tumor lies over the dorsal aspect of the dura [7]. Its port wine color or dark brown appearance contrasts well with the normal epidural fat [2]. Increased body weight and pregnancy appear to exacerbate the symptoms because of changes in the tumor mass and volume [2].

The first published case of spinal angiolipoma was described by Berenbruch in 1890 [8]: a 16-year-old boy with numerous cutaneous lipomas developed progressive paraparesis with hyper-reflexia, and was diagnosed at autopsy after unsuccessful surgical treatment. In 1974, Lin and Lin [9] categorized angiolipomas into two subtypes: non-infiltrating and infiltrating. The non-infiltrating type is more common and is usually well encapsulated. The infiltrating ones are partially or entirely unencapsulated, ill-defined, and invade the contiguous bone and adjacent soft tissues [10].

Surgery appears to be the treatment of choice, and the post-operative outcome after surgical management of this lesion is favorable. Most authors report good outcomes after surgical excision of spinal angiolipoma despite severe pre-operative neurological deterioration [11]. Complete excision appears to be curative in most cases [5,6], although most patients have a good prognosis, even with subtotal removal, because these lesions are slowly growing and do not undergo malignant transformation [10]. However, in cases with infiltration or extracanal extension to the thoracic cavity, a total resection can be more difficult. For infiltrating angiolipomas that involve the vertebral body rather than the posterior arch, total removal of the tumor and stabilization of the involved vertebral body using the anterolateral approach may be desirable [7,12]. However, some authors believe that the tumor-invaded vertebral body should be preserved because, analogous to vertebral hemangiomas, spinal angiolipomas may not enlarge [11]. Surgical management of infiltrating spinal angiolipoma remains under debate.

If complete removal is not easily achievable, wider resection, followed by radiotherapy, can be considered [1]. There are three reported cases that administered post-operative radiotherapy following a partial excision owing to concerns of potential malignancy [4,13,14]. Gelabert-Gonzalez and Garcia-Allut [6] reviewed the literature of spinal angiolipoma dating from 1892 and found 123 cases of spinal angiolipomas. They insisted that no adjuvant radiation should be applied to patients with spinal angiolipoma because the prognosis is very good, even in the patients with infiltrating tumors. However, a case of recurrence of an angiolipoma has been reported, even with successful initial surgery [15]. In our case, we applied radical resection combined with instrumented fixation. We successfully achieved total resection without adjuvant radiotherapy and our patient’s clinical symptoms improved after the surgery. She had no signs of tumor recurrence, neurological deficit, or post-operative scoliosis during the follow-up period.

## Conclusion

We describe a case of thoracic epidural angiolipoma treated by radical resection combined with instrumented spinal fixation. Spinal angiolipomas are rare but specific, and composed of varying proportions of mature fat cells and abnormal vascular elements. Surgical removal is the preferred treatment of spinal angiolipoma and the prognosis after surgical management is very good. Although outcomes remained favorable despite incomplete resections in a number of cases of spinal angiolipoma, complete removal is preferred. We successfully achieved total resection without any surgical complication by combining radical resection with instrumented spinal fixation.

## Consent

Written informed consent was obtained from the patient for publication of this case report and any accompanying images. A copy of the written consent is available for review by the Editor-in-Chief of this journal.

## Abbreviations

JOA: Japanese Orthopaedic Association; MMT: Manual muscle testing.

## Competing interests

The authors declare that they have no competing interests.

## Authors’ contributions

YN carried out the concept development, design, literature search, data collection, and writing. NS carried out the supervision and data collection (surgical and patient management). YT carried out data collection (patient management). AT carried out data collection (surgical and patient management). KN carried out data collection (surgical and patient management). YF carried out data collection (surgical and patient management). All authors read and approved the final manuscript.
